# Effects of prednisone on eosinophilic bronchitis in asthma: a systematic review and meta-analysis*,**

**DOI:** 10.1590/S1806-37132014000500012

**Published:** 2014

**Authors:** Thiago Mamôru Sakae, Rosemeri Maurici, Daisson José Trevisol, Marcia Margaret Menezes Pizzichini, Emílio Pizzichini

**Affiliations:** Graduate Program in Medical Sciences, Federal University of Santa Catarina, Florianópolis, Brazil; Graduate Program in Health Sciences, University of Southern Santa Catarina, Tubarão, Brazil; Graduate Program in Health Sciences, University of Southern Santa Catarina, Tubarão, Brazil; Graduate Program in Medical Sciences, Federal University of Santa Catarina, Florianópolis, Brazil; Graduate Program in Medical Sciences, Federal University of Santa Catarina, Florianópolis, Brazil

**Keywords:** Meta-analysis, Bronchitis, Asthma, Pulmonary eosinophilia, Evidence-based medicine, Prednisone

## Abstract

**OBJECTIVE:**

To evaluate the effect size of oral corticosteroid treatment on eosinophilic bronchitis in asthma, through systematic review and meta-analysis.

**METHODS:**

We systematically reviewed articles in the Medline, Cochrane Controlled Trials Register, EMBASE, and LILACS databases. We selected studies meeting the following criteria: comparing at least two groups or time points (prednisone vs. control, prednisone vs. another drug, or pre- vs. post-treatment with prednisone); and evaluating parameters before and after prednisone use, including values for sputum eosinophils, sputum eosinophil cationic protein (ECP), and sputum IL-5-with or without values for post-bronchodilator FEV_1_-with corresponding 95% CIs or with sufficient data for calculation. The independent variables were the use, dose, and duration of prednisone treatment. The outcomes evaluated were sputum eosinophils, IL-5, and ECP, as well as post-bronchodilator FEV_1_.

**RESULTS:**

The pooled analysis of the pre- vs. post-treatment data revealed a significant mean reduction in sputum eosinophils (↓8.18%; 95% CI: 7.69-8.67; p < 0.001), sputum IL-5 (↓83.64 pg/mL; 95% CI: 52.45-114.83; p < 0.001), and sputum ECP (↓267.60 µg/L; 95% CI: 244.57-290.63; p < 0.0001), as well as a significant mean increase in post-bronchodilator FEV_1_ (↑8.09%; 95% CI: 5.35-10.83; p < 0.001).

**CONCLUSIONS:**

In patients with moderate-to-severe eosinophilic bronchitis, treatment with prednisone caused a significant reduction in sputum eosinophil counts, as well as in the sputum levels of IL-5 and ECP. This reduction in the inflammatory response was accompanied by a significant increase in post-bronchodilator FEV_1_.

## Introduction

Eosinophilic bronchitis is a relatively new concept.^(^ 1 ^)^ The term was initially used in order to define the well-known allergic inflammatory response in asthma, characterized by an elevated number of eosinophils in tissues or bronchial secretions, typically in spontaneous or induced sputum. However, eosinophilia is neither specific to nor exclusive to asthma. Eosinophilic bronchitis has been reported in association with COPD,^(^ 2 ^)^ bronchiectasis,^(^ 2 ^,^ 3 ^)^ and chronic cough, with or without asthma.^(^ 1 ^-^ 3 ^)^ Nevertheless, eosinophilic bronchitis in asthma is relevant for various reasons: it precedes the clinical and physiological manifestations of asthma exacerbations induced by the withdrawal of corticosteroid treatment^(^ 4 ^,^ 5 ^)^; it has been associated with the risk of such exacerbations^(^ 4 ^-^ 8 ^)^; and a reduction in eosinophilia is a recognized marker of response to corticosteroid treatment.^(^ 1 ^,^ 9 ^)^

Predicting the response to corticosteroid treatment is relevant, particularly in asthma, because the suppression or attenuation of eosinophilic airway inflammation reduces the risk of subsequent exacerbations.^(^ 6 ^,^ 8 ^,^ 10 ^)^ Systemic corticosteroids are potent anti-inflammatory drugs and the most effective therapy for suppressing airway inflammation and eosinophilia.^(^ 8 ^,^ 10 ^)^ However, their long-term use is limited by side effects including osteoporosis, cataracts, and adrenal suppression. Currently, systemic corticosteroids are recommended to treat acute exacerbations of asthma, because they prevent the progression of exacerbations, decrease the hospitalization rate, reduce morbidity, and can be effective even when used for short periods of time.^(^ 11 ^)^ They are also used as an add-on therapy to treat severe eosinophilic asthma.^(^ 9 ^,^ 10 ^,^ 12 ^)^

Only a few, small studies have examined the effectiveness of using oral corticosteroids to reduce eosinophilic airway inflammation in asthma. We therefore aimed to examine the effect size of oral corticosteroids for the treatment of airway eosinophilia in asthma patients, through systematic review and meta-analysis. 

## Methods

### Search strategy

We searched the literature within the following electronic databases: the Cochrane Central Register of Controlled Trials (The Cochrane Library 2007, issue 4), which contains the Acute Respiratory Infections Group's Specialized Register; Medline (1966-2012); EMBASE (1974-2012); and LILACS (1982-2012). We conducted the following searches for terms in isolation or in combination (with Boolean operators):

1. "prednisone" OR "prednisolone"
2. "asthma" OR "bronchial asthma" OR "asthma exacerbation" OR "asthma exacerbations"
3. "bronchial hyperresponsiveness" OR "bronchial hyperreactivity"
4. "cytokines"
5. "induced sputum"
6. "asthma" OR "bronchial asthma" OR "asthma exacerbation" OR "asthma exacerbations" OR "bronchial hyperresponsiveness" OR "bronchial hyperreactivity"
7. "prednisone" OR "prednisolone" AND "asthma" OR "bronchial asthma" OR "asthma exacerbation" OR "asthma exacerbations" OR "bronchial hyperresponsiveness" OR "bronchial hyperreactivity"
8. "cytokines" OR "induced sputum"
9. "prednisone" OR "prednisolone" OR "asthma" OR "bronchial asthma" OR "asthma exacerbation" OR "asthma exacerbations" OR "bronchial hyperresponsiveness" OR "bronchial hyperreactivity" AND "cytokines" OR "induced sputum"

We also searched the bibliographic references of all of the articles thus selected, even if the former had not been identified in the database search.

### Eligibility criteria 

We initially selected articles meeting the following criteria: being a clinical trial of the effects of prednisone or prednisolone (in comparison with those of another treatment of eosinophilic bronchitis in asthma or versus a control) or a pre- and post-treatment study examining the effects of prednisone or prednisolone on eosinophilic bronchitis; involving treatment with prednisone or prednisolone for at least three days; and showing pre- and post-treatment outcomes that include sputum eosinophils, IL-5, and eosinophil cationic protein (ECP), as well as post-bronchodilator FEV_1_, with corresponding 95% CIs or with sufficient data for calculation. No limitations were set for participant ages or the definition of asthma severity as used in individual studies. No unpublished or ongoing studies were included.

Two of the authors of the present study, working individually, screened the titles and abstracts of identified citations and independently acquired the full text of any article that they judged potentially eligible. They also independently reviewed and selected trials from the search results, assessing the suitability, methodology, and quality of the studies. Cases of disagreement or uncertainty were resolved by consensus or by consulting one of the other authors. 

### Data extraction

We extracted the data using a protocol adapted from the Preferred Reporting Items for Systematic Reviews and Meta-Analyses statement,^(^ 13 ^)^ including the study identification data; the duration of the study; the study design; inclusion and exclusion criteria; criteria for asthma diagnosis; the age and gender of the participants; the number of participants; the randomization method; the severity of asthma in the study group(s); the methods of sputum processing and measurement; and the methods of evaluating post-treatment changes in sputum eosinophils, IL-5, and ECP, as well as post-bronchodilator FEV_1_.

### Statistical analysis

We analyzed the data using the MIX software for meta-analysis, version 1.7 (Kitasato Clinical Research Center, Sagamihara, Japan).^(^ 14 ^)^ We pooled the included studies to yield the means or medians of sputum eosinophils, ECP, IL-5, and FEV_1_, with the respective 95% CIs or SEs. For continuous variables, we calculated the means and 95% CIs. When the authors reported SDs, we used them to calculate SEs with the following formula: 

*SD* = SE * √^(N)^

When the SDs were not available for these variables, we transformed 95% CIs into SDs, using the following formula: 

*SE* = (*upper limit of 95% CI *−* lower limit of 95% CI *)/(1.96*2), *SD* = SE * √^(N)^

We quantified inconsistency among the pooled estimates with Higgins' I^2^ statistic, which measures the extent of true heterogeneity and is determined as follows: 

*I2* = [(*Q* − *df *)/*Q*] × 100

where *Q* is Cochran's Q (based on the chi-square statistic), and *df* is degrees of freedom. This illustrates the percentage variability in effect estimates resulting from heterogeneity rather than sampling error.^(^ 15 ^)^ If heterogeneity was found, we used a random-effects model. We performed sensitivity analyses comparing random-effects and fixed-effects models. We assessed potential for publication bias using Egger's test, Higgins' I^2^, and funnel plots. A random-effects model was used for the analysis of sputum eosinophils, IL-5, and ECP, because of the high heterogeneity of these markers among the studies, being Q = 168.1; p < 0.001; I^2^ = 96.4%, Q = 8.7; p = 0.013; I^2^ = 77.0%, and Q = 700.9; p < 0.001; I^2^ = 99.6%, respectively.

## Results

**Figure 1:**
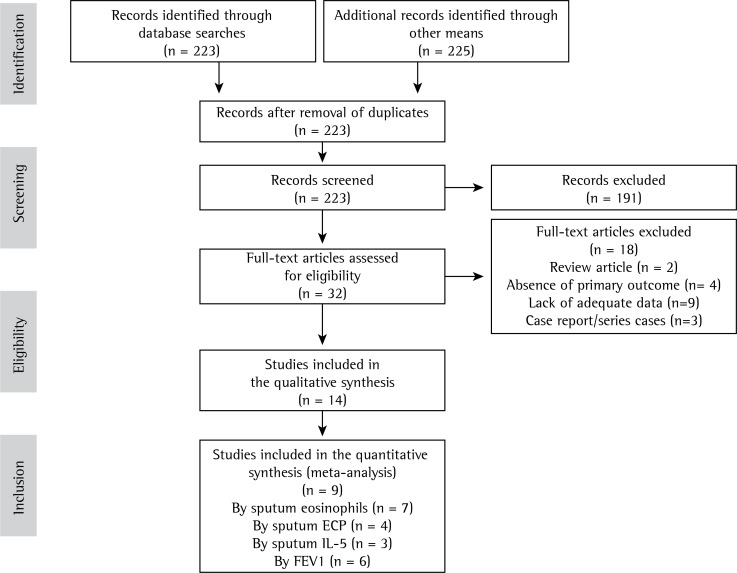
Flowchart of study selection. ECP: eosinophil cationic protein.

Through the database searches, we identified a total of 223 articles. Upon review of the titles and abstracts, we excluded 191 studies (Figure 1). Among the remaining 32 articles, some were further excluded: for lacking any information on primary outcomes (n = 4),^(^ 6 ^,^ 16 ^)^ for being a review article or meta-analysis (n = 2),^(^ 17 ^,^ 18 ^)^ for being a case report or case series (n = 3),^(^ 7 ^,^ 19 ^,^ 20 ^)^ or for lacking adequate data for the meta-analysis (n = 9; evaluating a different drug, lacking a control group, or lacking pre- and post-treatment data related to the use of prednisone or prednisolone). ^(^ 12 ^,^ 21 ^-^ 28 ^)^ We reviewed the remaining 14 articles and found that only 8 met the inclusion criteria.^(^ 8 ^,^ 10 ^,^ 29 ^-^ 34 ^)^ The characteristics of the included studies are presented in Table 1. 

**Table 1:** Characteristics of the studies included.

Reference	Characteristics of the study	Treatment	Pre-treatment vs. post-treatment
Baigelman et al.^(30)^	Design: observational	Drug: prednisone	Sputum eosinophils, mean ± SD: 26.0 ± 19.0% vs. 8.0 ± 5.0%
	Sample: 11 asthma patients (3 males and 8 females; 29-60 years of age) followed for at least 4 years	Dose: 80 mg/day	Sputum ECP: [not measured]
			post-bronchodilator FEV_1_, mean ± SD: 1.03 ± 0.41 L vs. 1.32 ± 0.51 L
	Profile: asthma exacerbation	Duration: 3 days	IL-5: [not measured]
Claman et al.^(31)^	Design: randomized controlled trial		
	Sample: 24 asthma patients—oral prednisone group (n = 12, 7 males and 5 females) and placebo group (n = 12, 6 males and 6 females)—excluding patients having used inhaled or oral corticosteroids in the last 6 weeks, having had an upper respiratory infection in the last 6 weeks, and having a smoking history of > 10 pack-years	Drug: oral prednisone	Sputum eosinophils, mean ± SE: 14.1 ± 5.0% vs. 1.8 ± 0.8%
		Dose: 0.5 mg/kg per day	Sputum ECP, mean ± SE: 325 ± 131 µg/mL vs. 144 ± 84 µg/mL
	Profile: asthma exacerbation	Duration: 6 days	post-bronchodilator FEV_1_, mean ± SD: 88 ± 5.2% vs. 91 ± 4.87%
			IL-5: [not measured]
Keatings et al.^(34)^	Design: single-blind crossover study	Drug: prednisolone	Sputum eosinophils, mean ± SD: 6.66 ± 0.98% vs. 0.99 ± 0.25%
			Sputum ECP, mean ± SD: 687.0 ± 87.0 µg/L vs. 80.2 ± 14.2 µg/L
	Sample: 15 COPD patients (excluded from this meta-analysis) and 11 patients with mild atopic asthma (1 excluded; 10 included for analysis; mean age 29.8 ± 3.4 years)	Dose: 30 mg/day	
		Duration: 2 weeks	post-bronchodilator FEV_1_:
	Profile: stable asthma		-in %, mean ± SD: 95.9 ± 5.7% vs. [not described]
			-in L, mean ± SE: [not described] vs. 3.92 ± 0.32 L
			IL-5: [not measured]
Pizzichini et al.^(8)^	Design: observational	Drug: prednisone	Sputum eosinophils, mean ± SD: 17.6 ± 10.0% vs. 0.89 ± 0.90%
	Sample: 10 asthma patients	Dose: 30 mg/day for 5 days, tapered to zero by day 10	Sputum ECP, mean ± SD: 5,338.4 ± 6690.7 µg/L vs. 985.5 ± 1311.0 µg/L
	Profile: asthma exacerbation	Duration: 10 days	post-bronchodilator FEV_1_, mean ± SD: 1.5 ± 0.3 L vs. 2.5 ± 0.5 L
			IL-5, mean ± SD: 201 ± 128 pg/mL vs. 0.0 ± 55.6 pg/mL
Pizzichini et al.^(10)^	Design: observational	Drug: prednisone	Sputum eosinophils, mean ± SD: 16.3 ± 32.3% vs. 0.0 ± 0.5%
	Sample: 8 patients with prednisone-dependent asthma; >12% variability in FEV_1_, baseline mean, 18.5% (range, 13-27%)	Dose: 30 mg/day	Sputum ECP, mean ± SD: 7480 ± 5240 µg/L vs. 700 ± 784 µg/L
	Profile: severe asthma	Duration: 7 days	post-bronchodilator FEV_1_, mean ± SD: 55.7 ± 6.84% vs. 80.0 ± 15.91%
			IL-5, mean ± SD: 66.5 ± 150 pg/mL vs. 44.1 ± 86 pg/mL
Di Franco et al.^(32)^	Design: randomized controlled trial	Drug: prednisone	Sputum eosinophils: 52.0% [no SD or SE] vs. 11.0% [no SD or SE]
	Sample: 40 adult nonsmokers (9 males and 31 females; mean age, 45 ± 13 years; 3 excluded), in two arms—fluticasone (1,000 µg/day; n = 18) and prednisone (n = 19)	Dose: 40 mg/day, tapered to 10 mg/day by reducing the dose by 5 mg every other day	Sputum ECP: 904 µg/L [no SD or SE] vs. [not described]
	Profile: asthma exacerbation	Duration: 6 days	post-bronchodilator FEV_1_, mean ± SD: 51.5 ± 14.4% vs. 83.6 ± 21.1%
			IL-5: [not measured]
Scheicher et al.^(33)^	Design: controlled observational	Drug: oral prednisone	-
	Sample: 51 subjects—21 normal subjects and 30 patients with asthma (13 males and 17 females; mean age, 41 years), 9 of whom were steroid-naïve—the 9 steroid-naïve patients receiving oral prednisone and being evaluated before and after the treatment	Dose: 40 mg/day	
	Profile: stable asthma	Duration: 14 days	
Dente et al.^(29)^	Design: randomized controlled trial	Drug: oral prednisone	-
	Sample: 59 patients with severe refractory asthma, randomized to receive prednisone (n = 39) or placebo (n = 20)	Dose: 0.5 mg/kg per day	
	Profile: severe asthma	Duration: 2 weeks	

ECP: eosinophil cationic protein.

### Effects on sputum eosinophils, IL-5, and ECP

**Figure 2:**
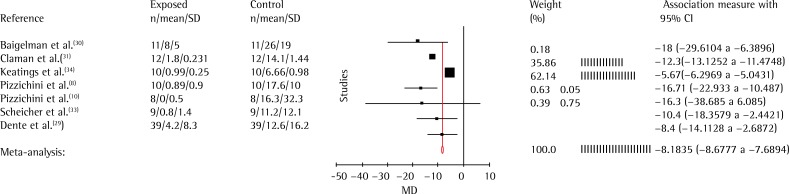
Reduction in sputum eosinophils after treatment with oral prednisone or prednisolone.

**Figure 3:**
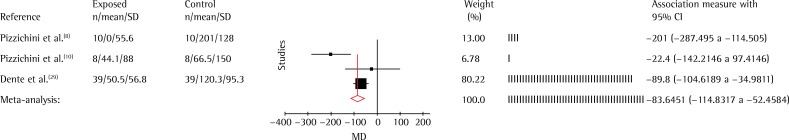
Reduction in sputum IL-5 levels after treatment with prednisone.

**Figure 4:**
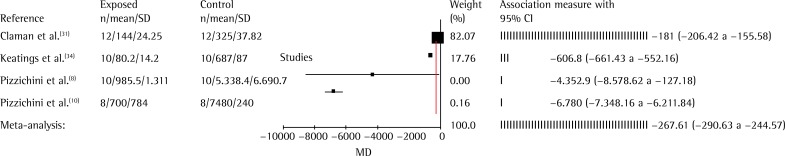
Reduction in sputum levels of eosinophil cationic protein after treatment with oral prednisone or prednisolone.

The pooled analysis (n = 198) showed a six-fold mean reduction in the number of sputum eosinophils after treatment (↓8.2%; 95% CI: 7.7-8.7; p < 0.001; Figure 2). Among studies evaluating IL-5 (in pg/mL) before and after treatment with prednisone or prednisolone (n = 114), there was an approximately four-fold mean decrease in IL-5 levels (↓83.6; 95% CI: 52.5-83.6; p < 0.001; Figure 3). In addition, among the studies evaluating ECP (in µg/L) in subjects receiving prednisone or prednisolone (n = 80), the treatment resulted in a five-fold mean reduction in ECP levels (↓267.6; 95% CI: 244.6-290.6; p < 0.001; Figure 4). 

### Effects on FEV_1_

**Figure 5:**
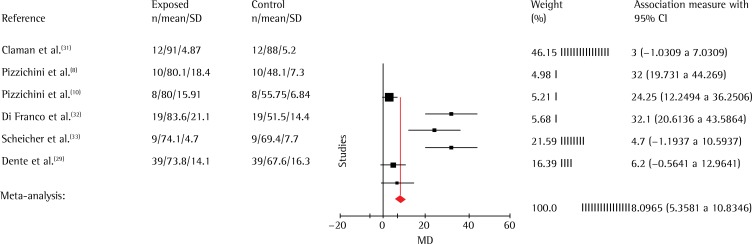
Increase in post-bronchodilator FEV1 (% of predicted) after treatment with prednisone.

We also analyzed changes in post-bronchodilator FEV_1_ after treatment with prednisone or prednisolone in the 194 asthma patients for whom the relevant data were available. ^(^ 8 ^,^ 10 ^,^ 29 ^-^ 32 ^)^ In that analysis, we also used a random-effects model, because of the high heterogeneity (Q = 46.03; p < 0.0001; I^2^ = 89.1%). After 6-14 days of treatment, there was a significant mean increase in post-bronchodilator FEV_1_ (↑8.1%; 95% CI: 5.3-10.8; z = 5.8; p < 0.001; Figure 5). An analysis of the data regarding the absolute values for post-bronchodilator FEV_1_ (in liters) showed a mean post-treatment increase, from 1.88 to 2.34 L (↑0.46 L; p < 0.001; data not shown). 

### Management of results

**Figure 6:**
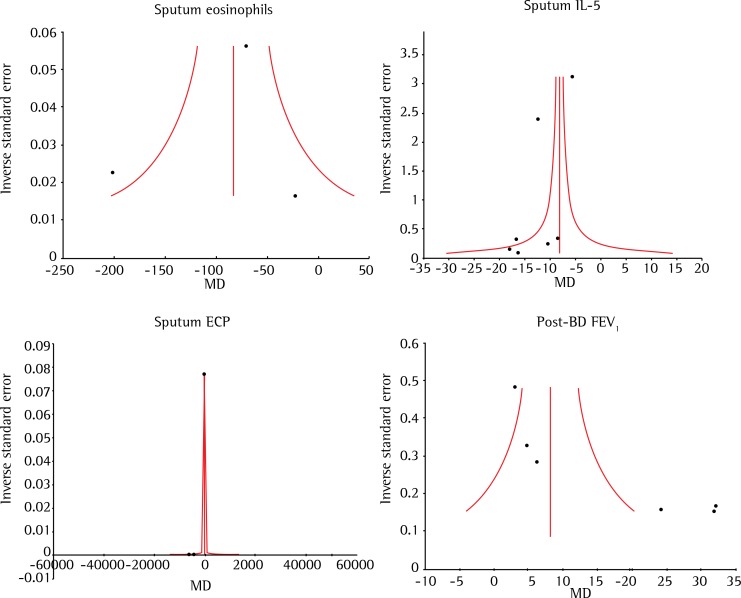
plots. MD: median; ECP: eosinophil cationic protein; and BD: bronchodilator.

Because of the high heterogeneity, we conducted a meta-regression to examine the effects of treatment with prednisone or prednisolone by age, gender, and dose (Figure 6). The prednisone dose appeared to be responsible for the heterogeneity in sputum eosinophil counts (T^2^ = 8.753) and ECP (T^2^ = 172.8). Linear regression did not show an association between prednisone dose and sputum eosinophils (p = 0.55), sputum ECP (p = 0.38), sputum IL-5 (p = 1.00) or post-bronchodilator FEV_1_ (p = 0.27).

## Discussion

Our analyses show that treatment with prednisone or prednisolone is highly effective in reducing sputum eosinophils in eosinophilic bronchitis. This is accompanied by a reduction in other sputum inflammatory markers linked to eosinophilic bronchitis, such as ECP and IL-5. In addition, treatment of eosinophilic bronchitis with prednisone or prednisolone was shown to effect a significant increase in post-bronchodilator FEV_1_. 

Data suggest that sputum eosinophilia and high levels of eosinophilic markers are associated with poor asthma control rather than with the severity of asthma.^(^ 7 ^,^ 8 ^,^ 21 ^,^ 26 ^)^ The corticosteroid-responsive component of asthma is eosinophilic bronchitis, which can now be recognized by the reliable method of counting cells in induced sputum samples.^(^ 8 ^)^ Adequate treatment with corticosteroids reduces the proportion of eosinophils in sputum to within the normal range, even in prednisone-dependent asthma.^(^ 8 ^,^ 19 ^)^ In this meta-analysis, there was a six-fold reduction in sputum eosinophils after treatment with prednisone or prednisolone.

Because a reduction in sputum eosinophilia is associated with a positive clinical and functional response to corticosteroids, our results support the recommendation to increase the dose of corticosteroids when asthma becomes uncontrolled. ^(^ 8 ^,^ 21 ^)^ In a multiple regression analysis, ten Brinke et al.^(^ 23 ^)^ found that the most important factor potentially associated with persistent airflow limitation in severe asthma was a proportion of eosinophils in sputum > 2% (adjusted OR = 7.7). However, there are individuals with uncontrolled asthma who do not present with sputum eosinophilia. Whether that subgroup could be less responsive to corticosteroids remains to be established.

Eosinophils have long been regarded as key inflammatory mediators in the pathogenesis of asthma, although their exact role is unclear. In studies of heterogeneous populations of subjects with asthma, the downregulation of eosinophil activity via targeted inhibition of IL-5 (a pro-eosinophilic cytokine) has yielded disappointing results.^(^ 35 ^)^

In one study of patients with severe asthma and refractory eosinophilic airway inflammation,^(^ 22 ^)^ intramuscular administration of triamcinolone was found to reduce the mean proportion of eosinophils in sputum from 12.6% to 0.2%, similar to the reductions achieved through the use of inhaled corticosteroids (> 1,600 µg/day) or chronic oral prednisone.^(^ 29 ^,^ 32 ^)^ The authors also observed an increase in FEV_1_ and a reduction in the use of rescue medication.^(^ 22 ^)^ These data are in agreement with those of other studies of asthma patients, confirming that sputum eosinophilia is a good predictor of the response to corticosteroids.^(^ 6 ^-^ 8 ^,^ 36 ^)^ The reason for this distinct short-term effect of corticosteroids, as opposed to the well-known, positive, long-term effect of corticosteroids in most asthma patients, is not known.^(^ 29 ^)^ One possible explanation is that non-eosinophilic inflammation might respond to corticosteroids more slowly than does eosinophilic inflammation.^(^ 37 ^)^

The data presented here support the usefulness of sputum eosinophil assessment in predicting when patients with severe asthma might benefit from an increase in the dose of corticosteroids. ^(^ 6 ^-^ 8 ^,^ 29 ^,^ 36 ^)^ Classifying severe asthma phenotypes as eosinophilic and non-eosinophilic might have clinical implications for the choice of pharmacological therapy.^(^ 7 ^,^ 19 ^,^ 29 ^)^

Persistence of eosinophilia in severe asthma could be a reflection of corticosteroid insensitivity, and refractory asthma might respond to the use of anti-IL-5 therapy with mepolizumab. ^(^ 4 ^,^ 5 ^,^ 38 ^,^ 39 ^)^ As previously mentioned, we found that sputum IL-5 levels decreased after treatment with prednisone or prednisolone. Because IL-5 is a pro-inflammatory cytokine that increases the recruitment, activation, and survival of eosinophils, it is considered of pivotal importance in the pathophysiology of asthma.^(^ 40 ^)^ Ying et al.^(^ 41 ^)^ showed that IL-5 is highly expressed in T cells, eosinophils, and mast cells in bronchial biopsy specimens collected from patients with asthma. Other studies have shown that sputum levels of IL-5 trend higher in patients with eosinophilic asthma, whereas those of IL-8 trend higher in patients with non-eosinophilic asthma. ^(^ 29 ^,^ 42 ^)^ There is evidence that IL-5 is detectable in the induced sputum of asthma patients and that sputum levels of IL-5 are higher in patients with severe asthma than in those with mild-to-moderate asthma.^(^ 10 ^,^ 42 ^)^

Our results show that systemic corticosteroids are effective not only in reducing sputum eosinophil counts but also in inhibiting the release of pro-inflammatory cytokines that play a relevant role in perpetuating airway inflammation in patients with refractory asthma. Our data agree with those of other studies showing that corticosteroids decrease the number of activated T cells expressing messenger RNA of IL-4 and IL-5 in the BAL fluid of asthma patients, regardless of the severity of the asthma.^(^ 43 ^)^ Our results also show that treatment with prednisone or prednisolone can effect a four-fold reduction in sputum ECP levels. When we considered the ECP levels in sputum supernatants, we found that those levels were associated with poor asthma control, further underscoring the fact that eosinophils play a role in this equation.^(^ 28 ^)^ However, despite expectations that patients with severe asthma would show higher sputum ECP levels, Romagnoli et al.^(^ 21 ^)^ observed no differences among groups of asthma patients, stratified by asthma severity, in terms of the sputum levels of ECP.

In a study of acute exacerbations of asthma, Baigelman et al.^(^ 30 ^)^ demonstrated that FEV_1_ improves within the first 24 h of treatment with prednisone or prednisolone, further improvement being observed after 48-72 h of such treatment. However, in a similar study, Belda et al.^(^ 44 ^)^ found no change in FEV_1_ in the first 24 h. Aggarwal & Bhoi^(^ 12 ^)^ also studied acute exacerbations of asthma and suggested that intravenous methylprednisolone followed by oral methylprednisolone is a more efficacious and safer treatment regimen than is intravenous hydrocortisone followed by oral prednisolone. However, those authors employed clinical and spirometric evaluation alone, without analyzing inflammatory mediators in sputum or other respiratory secretions.

In a meta-analysis conducted in 1992, Rowe et al.^(^ 17 ^)^ showed that the use of corticosteroids early in the treatment of asthma exacerbations reduces the number of hospital admissions in adults and children, as well as showing that corticosteroids are effective in preventing relapse in the outpatient treatment of asthma exacerbations. Oral and intravenous corticosteroids appear to have equivalent effects on pulmonary function in acute exacerbations,^(^ 17 ^)^ and recent studies have shown that inhaled and oral corticosteroids are also equally effective.^(^ 24 ^,^ 32 ^,^ 33 ^,^ 44 ^)^

Despite the heterogeneity among the studies evaluated here, in terms of the doses and duration of treatment/follow-up,^(^ 8 ^,^ 10 ^,^ 29 ^-^ 31 ^)^ most of the exposure to prednisone or prednisolone was at > 30 mg/day, which is sufficient to suppress sputum eosinophils, reduce ECP levels, and inhibit IL-5, as well as to increase FEV_1_. However, because of the high degree of heterogeneity, it was necessary to perform a meta-regression to adjust for dose and duration of treatment.

Meta-analysis is a powerful tool for studying cumulative data from individual studies with small sample sizes and low statistical power. Pooling effects from individual studies through meta-analysis can increase the statistical power and can help detect modest differences in risk among study groups.

It is possible to achieve a detectable change in inflammatory indices during a 14-day course of treatment with corticosteroids. Clinical benefits and anti-inflammatory effects have been reported in asthma patients treated with such short regimens, which are commonly used in clinical practice.^(^ 12 ^,^ 34 ^)^

In conclusion, we found that, in patients with moderate-to-severe eosinophilic bronchitis, treatment with prednisone or prednisolone effected a significant reduction in sputum eosinophil counts, as well as in the sputum levels of IL-5 and ECP. This reduction in the inflammatory response was accompanied by a significant increase in post-bronchodilator FEV_1_.

## References

[1] Kelly MM, Leigh R, Jayaram L, Goldsmith CH, Parameswaran K, Hargreave FE (2006). Eosinophilic bronchitis in asthma: a model for
establishing dose-response and relative potency of inhaled
corticosteroids. J Allergy Clin Immunol.

[2] Hargreave FE, Parameswaran K (2006). Asthma, COPD and bronchitis are just components of
airway disease. Eur Respir J.

[3] Hargreave FE, Leigh R, Parameswaran K (2006). Asthma as a disease concept. Lancet.

[4] Haldar P, Brightling CE, Hargadon B, Gupta S, Monteiro W, Souza A (2009). Mepolizumab and exacerbations of refractory eosinophilic
asthma. N Engl J Med.

[5] Nair P, Pizzichini MM, Kjarsgaard M, Inman MD, Efthimiadis A, Pizzichini E (2009). Mepolizumab for prednisone-dependent asthma with sputum
eosinophilia. N Engl J Med.

[6] Jayaram L, Pizzichini MM, Cook RJ, Boulet LP, Lemière C, Pizzichini E (2006). Determining asthma treatment by monitoring sputum cell
counts: effect on exacerbations. Eur Respir J.

[7] Parameswaran K, Pizzichini MM, Li D, Pizzichini E, Jeffery PK, Hargreave FE (1998). Serial sputum cell counts in the management of chronic
airflow limitation. Eur Respir J.

[8] Pizzichini MM, Pizzichini E, Clelland L, Efthimiadis A, Mahony J, Dolovich J (1997). Sputum in severe exacerbations of asthma: kinetics of
inflammatory indices after prednisone treatment. Am J Respir Crit Care Med.

[9] Nolte H, Pavord I, Backer V, Spector S, Shekar T, Gates D (2013). Dose-dependent anti-inflammatory effect of inhaled
mometasone furoate/formoterol in subjects with asthma. Respir Med.

[10] Pizzichini MM, Pizzichini E, Clelland L, Efthimiadis A, Pavord I, Dolovich J (1999). Prednisone-dependent asthma: inflammatory indices in
induced sputum. Eur Respir J.

[11] Kroegel C (2009). Global Initiative for Asthma (GINA) guidelines: 15 years
of application. Expert Rev Clin Immunol.

[12] Aggarwal P, Bhoi S (2010). Comparing the efficacy and safety of two regimens of
sequential systemic corticosteroids in the treatment of acute exacerbation of
bronchial asthma. J Emerg Trauma Shock.

[13] Liberati A, Altman DG, Tetzlaff J, Mulrow C, Gøtzsche PC, Ioannidis JP (2009). The PRISMA statement for reporting systematic reviews
and meta-analyses of studies that evaluate health care interventions: explanation
and elaboration. PLoS Med.

[14] Bax L, Yu LM, Ikeda N, Tsuruta H, Moons KG (2006). Development and validation of MIX: comprehensive free
software for meta-analysis of causal research data. BMC Med Res Methodol.

[15] Higgins JPT, Green S (2005). Highly sensitive search strategies for identifying
reports of randomized controlled trials in MEDLINE. Cochrane Handbook for Systematic Reviews of Interventions
version 4.2.5. Chichester,.

[16] Jayaram L, Duong M, Pizzichini MM, Pizzichini E, Kamada D, Efthimiadis A (2005). Failure of montelukast to reduce sputum eosinophilia in
high-dose corticosteroid-dependent asthma. Eur Respir J.

[17] Rowe BH, Keller JL, Oxman AD (1992). Effectiveness of steroid therapy in acute exacerbations
of asthma: a meta-analysis. Am J Emerg Med.

[18] Bousquet J, Ben-Joseph R, Messonnier M, Alemao E, Gould AL (2002). A meta-analysis of the dose-response relationship of
inhaled corticosteroids in adolescents and adults with mild to moderate persistent
asthma. Clin Ther.

[19] Parameswaran K, Leigh R, Hargreave FE (1999). Sputum eosinophil count to assess compliance with
corticosteroid therapy in asthma. J Allergy Clin Immunol.

[20] Wong AG, Pavord ID, Sears MR, Hargreave FE (1996). A case for serial examination of sputum inflammatory
cells. Eur Respir J.

[21] Romagnoli M, Vachier I, Tarodo de la Fuente P, Meziane H, Chavis C, Bousquet J (2002). Eosinophilic inflammation in sputum of poorly controlled
asthmatics. Eur Respir J.

[22] ten Brinke A, Zwinderman AH, Sterk PJ, Rabe KF (2004). Bel EH "Refractory" eosinophilic airway inflammation in
severe asthma: effect of parenteral corticosteroids.. Am J Respir Crit Care Med.

[23] ten Brinke A, Zwinderman AH, Sterk PJ, Rabe KF, Bel EH (2001). Factors associated with persistent airflow limitation in
severe asthma. Am J Respir Crit Care Med.

[24] van den Berge M, Arshad SH, Ind PW, Magnussen H, Hamelmann E, Kanniess F (2009). Similar efficacy of ciclesonide versus prednisolone to
treat asthma worsening after steroid tapering. Respir Med.

[25] van den Berge M, Kerstjens HA, Meijer RJ, de Reus DM, Koëter GH, Kauffman HF (2001). Corticosteroid-induced improvement in the PC20 of
adenosine monophosphate is more closely associated with reduction in airway
inflammation than improvement in the PC20 of methacholine. Am J Respir Crit Care Med.

[26] Xu J, Jiang F, Nayeri F, Zetterstrom O (2007). Apoptotic eosinophils in sputum from asthmatic patients
correlate negatively with levels of IL-5 and eotaxin. Respir Med.

[27] Kerzerho J, Wunsch D, Szely N, Meyer HA, Lurz L, Röse L (2012). Effects of systemic versus local administration of
corticosteroids on mucosal tolerance. J Immunol.

[28] Kulkarni NS, Hollins F, Sutcliffe A, Saunders R, Shah S, Siddiqui S (2010). Eosinophil protein in airway macrophages: a novel
biomarker of eosinophilic inflammation in patients with asthma. J Allergy Clin Immunol..

[29] Dente FL, Bacci E, Bartoli ML, Cianchetti S, Costa F, Di Franco A (2010). Effects of oral prednisone on sputum eosinophils and
cytokines in patients with severe refractory asthma. Ann Allergy Asthma Immunol.

[30] Baigelman W, Chodosh S, Pizzuto D, Cupples LA (1983). Sputum and blood eosinophils during corticosteroid
treatment of acute exacerbations of asthma. Am J Med.

[31] Claman DM, Boushey HA, Liu J, Wong H, Fahy JV (1994). Analysis of induced sputum to examine the effects of
prednisone on airway inflammation in asthmatic subjects. J Allergy Clin Immunol.

[32] Di Franco A, Bacci E, Bartoli ML, Cianchetti S, Dente FL, Taccola M (2006). Inhaled fluticasone propionate is effective as well as
oral prednisone in reducing sputum eosinophilia during exacerbations of asthma
which do not require hospitalization. Pulm Pharmacol Ther.

[33] Scheicher ME, Teixeira MM, Cunha FQ, Teixeira AL, Jr, Filho JT, Vianna EO (2007). Eotaxin-2 in sputum cell culture to evaluate asthma
inflammation. Eur Respir J.

[34] Keatings VM, Jatakanon A, Worsdell YM, Barnes PJ (1997). Effects of inhaled and oral glucocorticoids on
inflammatory indices in asthma and COPD. Am J Respir Crit Care Med.

[35] Barratt S (2009). Mepolizumab in corticosteroid-resistant eosinophilic
asthma. Thorax.

[36] Bacci E, Cianchetti S, Bartoli ML, Dente FL, Di Franco A, Vagaggini B (2006). Low sputum eosinophils predict the lack of response to
beclomethasone in symptomatic asthmatic patients. Chest.

[37] Barnes PJ (2006). Corticosteroids: the drugs to beat. Eur J Pharmacol.

[38] Chung KF (2012). Inflammatory biomarkers in severe asthma. Curr Opin Pulm Med.

[39] Antoniu SA (2009). Mepolizumab for difficult-to-control asthma with
persistent sputum eosinophilia. Expert Opin Investig Drugs.

[40] O'Byrne PM, Inman MD, Parameswaran K (2001). The trials and tribulations of IL-5, eosinophils, and
allergic asthma. J Allergy Clin Immunol..

[41] Ying S, Humbert M, Barkans J, Corrigan CJ, Pfister R, Menz G (1997). Expression of IL-4 and IL-5 mRNA and protein product by
CD4+ and CD8+ T cells, eosinophils, and mast cells in bronchial biopsies obtained
from atopic and nonatopic (intrinsic) asthmatics. J Immunol.

[42] Dente FL, Carnevali S, Bartoli ML, Cianchetti S, Bacci E, Di Franco A (2006). Profiles of proinflammatory cytokines in sputum from
different groups of severe asthmatic patients. Ann Allergy Asthma Immunol.

[43] Robinson DS, Hamid Q, Ying S, Tsicopoulos A, Barkans J, Bentley AM (1992). Predominant TH2-like bronchoalveolar T-lymphocyte
population in atopic asthma. N Engl J Med.

[44] Belda J, Margarit G, Martinez C, Bellido-Casado J, Casan P, Torrejón M (2007). Anti-inflammatory effects of high-dose inhaled
fluticasone versus oral prednisone in asthma exacerbations. Eur Respir J.

