# Supplementary material for: Effects of prednisone on eosinophilic bronchitis in asthma: a systematic review and meta-analysis
Source: J Bras Pneumol. 2014 Sep-Oct;40(5):552–63. doi: 10.1590/S1806-37132014000500012 (PMC4263337; doi:10.1590/S1806-37132014000500012)
Supplement: Translation

# Translation: Efeitos da prednisona na bronquite eosinofílica na asma: revisão sistemática e meta-análise*

**Authors:** Thiago Mamôru Sakae, Rosemeri Maurici, Daisson José Trevisol, Marcia Margaret Menezes Pizzichini, Emílio Pizzichini

**Affiliations:** Programa de Pós-Graduação em Ciências Médicas, Universidade Federal de Santa Catarina, Florianópolis (SC) Brasil; Programa de Pós-Graduação em Ciências da Saúde, Universidade do Sul de Santa Catarina, Tubarão (SC) Brasil; Programa de Pós-Graduação em Ciências da Saúde, Universidade do Sul de Santa Catarina, Tubarão (SC) Brasil; Programa de Pós-Graduação em Ciências Médicas, Universidade Federal de Santa Catarina, Florianópolis (SC) Brasil; Programa de Pós-Graduação em Ciências Médicas, Universidade Federal de Santa Catarina, Florianópolis (SC) Brasil

**Keywords:** Metanálise, Bronquite, Asma, Eosinofilia pulmonar, Medicina baseada em evidências, Prednisona

## Abstract

**OBJETIVO:**

Avaliar o tamanho do efeito do tratamento com prednisona oral na bronquite eosinofílica na asma por meio de revisão sistemática e meta-análise.

**MÉTODOS:**

Revisão sistemática de artigos nas bases de dados do Medline, *Cochrane Controlled Trials Register*, EMBASE e LILACS. Foram selecionados estudos que preencheram os seguintes critérios: comparar ao menos dois grupos ou dois momentos (prednisona vs. controle, prednisona vs. outra droga ou pré vs. pós-tratamento com prednisona) e avaliar parâmetros antes e depois do uso de prednisona, incluindo eosinófilos, proteína catiônica eosinofílica (PCE) e IL-5 no escarro - com ou sem valores de VEF_1_ pós-broncodilatador - com os IC95% correspondentes ou com dados suficientes para calculá-los. As variáveis independentes foram uso e dose de prednisona e duração do tratamento. Os desfechos avaliados foram eosinófilos, IL-5 e PCE no escarro, bem como VEF_1_ pós-broncodilatador.

**RESULTADOS:**

A análise conjunta dos dados de pré e pós-tratamento revelou uma redução significativa nas médias de eosinófilos no escarro (↓8,18%; IC95%: 7,69-8,67; p < 0,001), IL-5 no escarro (↓83,64 pg/mL; IC95%: 52,45-114,83; p < 0,001), PCE no escarro (↓267,60 μg/L; IC95%: 244,57-290,93; p < 0,001), assim como um aumento significativo na média de VEF_1_ pós-broncodilatador (↑8,09%; IC95%: 5,35-10,83; p < 0,001).

**CONCLUSÕES:**

Em pacientes com bronquite eosinofílica de moderada a grave, o tratamento com prednisona determinou uma redução significativa nos níveis de eosinófilos no escarro, assim como nos níveis de IL-5 e PCE no escarro. Essa redução na resposta inflamatória foi acompanhada de um aumento significativo do VEF_1_ pós-broncodilatador.

## Introdução

Bronquite eosinofílica é um conceito relativamente novo.^(^ 1 ^)^ O termo foi inicialmente usado para definir a resposta inflamatória alérgica na asma, que é bem conhecida e é caracterizada por um elevado número de eosinófilos em tecidos ou secreções brônquicas, tipicamente no escarro espontâneo ou induzido. No entanto, a eosinofilia não é nem específica nem exclusiva da asma. Há relatos de bronquite eosinofílica associada a DPOC,^(^ 2 ^)^ bronquiectasia^(^ 2 ^,^ 3 ^)^ e tosse crônica, com ou sem asma.^(^ 1 ^-^ 3 ^)^ Não obstante, a bronquite eosinofílica na asma é relevante por vários motivos: ela precede as manifestações clínicas e fisiológicas das exacerbações da asma induzidas pela interrupção do tratamento com corticosteroides^(^ 4 ^,^ 5 ^)^; há relatos de que está relacionada com o risco de exacerbações da asma^(^ 4 ^-^ 8 ^)^ e uma redução da eosinofilia é um marcador reconhecido de resposta ao tratamento com corticosteroides.^(^ 1 ^,^ 9 ^)^

É relevante prever a resposta ao tratamento com corticosteroides, particularmente na asma, pois a supressão ou atenuação da inflamação eosinofílica das vias aéreas reduz o risco de exacerbações subsequentes.^(^ 6 ^,^ 8 ^,^ 10 ^)^ Os corticosteroides sistêmicos são anti-inflamatórios potentes e a terapia mais eficaz para suprimir a inflamação das vias aéreas e a eosinofilia.^(^ 8 ^,^ 10 ^)^ No entanto, efeitos colaterais como osteoporose, catarata e supressão adrenal restringem o uso prolongado de corticosteroides sistêmicos. Atualmente, recomenda-se o uso de corticosteroides sistêmicos para tratar exacerbações agudas da asma, pois eles evitam a progressão das exacerbações, diminuem a taxa de hospitalização, reduzem a morbidade e podem ser eficazes mesmo quando usados durante curtos períodos.^(^ 11 ^)^ Eles também são usados como terapia adjuvante para asma eosinofílica grave.^(^ 9 ^,^ 10 ^,^ 12 ^)^

Apenas alguns estudos pequenos examinaram a eficácia do uso de corticosteroides orais para reduzir a inflamação eosinofílica das vias aéreas na asma. Nosso objetivo, portanto, foi examinar, por meio de revisão sistemática e meta-análise, o tamanho do efeito de corticosteroides orais para o tratamento de eosinofilia nas vias aéreas em pacientes com asma. 

## Métodos

### Estratégia de busca

Pesquisamos a literatura nos seguintes bancos de dados eletrônicos: *Cochrane Central Register of Controlled Trials* (*The Cochrane Library* 2007, número 4), que contém o *Acute Respiratory Infections Group's Specialized Register*; Medline (1966-2012); EMBASE (1974-2012) e LILACS (1982-2012). 

Realizamos as seguintes buscas por termos isolados ou combinados (com operadores booleanos):

1. "*prednisone*" *OR* "*prednisolone*"
2. "*asthma*" *OR* "*bronchial asthma*" *OR* "*asthma exacerbation*" *OR* "*asthma exacerbations*"
3. "*bronchial hyperresponsiveness*" *OR* "*bronchial hyperreactivity*"
4. "*cytokines*"
5. "*induced sputum*"
6. "*asthma*" *OR* "*bronchial asthma*" *OR* "*asthma exacerbation*" *OR* "*asthma exacerbations*" *OR* "*bronchial hyperresponsiveness*" *OR* "*bronchial hyperreactivity*"
7. "*prednisone*" *OR* "*prednisolone*" *AND* "*asthma*" *OR* "*bronchial asthma*" *OR* "*asthma exacerbation*" *OR* "*asthma exacerbations*" *OR* "*bronchial hyperresponsiveness*" *OR* "*bronchial hyperreactivity*"
8. "*cytokines*" *OR* "*induced sputum*"
9. "*prednisone*" *OR* "*prednisolone*" *OR* "*asthma*" *OR* "*bronchial asthma*" *OR* "*asthma exacerbation*" *OR* "*asthma exacerbations*" *OR* "*bronchial hyperresponsiveness*" *OR* "*bronchial hyperreactivity*" *AND* "*cytokines*" *OR* "*induced sputum*"

Também pesquisamos as referências bibliográficas de todos os artigos assim selecionados, mesmo que não tivessem sido identificadas na busca realizada nos bancos de dados. 

### Critérios de elegibilidade

Foram inicialmente selecionados artigos que preenchessem os seguintes critérios: ensaio clínico sobre os efeitos da prednisona ou prednisolona (em comparação com os efeitos de outro tratamento de bronquite eosinofílica em asma ou versus um controle) ou estudo pré e pós-tratamento examinando os efeitos da prednisona ou prednisolona na bronquite eosinofílica; estudo envolvendo o tratamento com prednisona ou prednisolona durante pelo menos três dias e estudo com desfechos pré e pós-tratamento que incluíssem eosinófilos, IL-5 e proteína catiônica eosinofílica (PCE) no escarro, bem como VEF_1_ pós-broncodilatador, com os IC95% correspondentes ou com dados suficientes para calculá-los. Não foram impostas restrições à idade dos participantes ou à definição de gravidade da asma em estudos individuais. Não foram incluídos estudos não publicados ou em andamento. 

Dois dos autores do presente estudo, trabalhando individualmente, fizeram uma triagem dos títulos e resumos de citações identificadas e independentemente adquiriram o texto completo de qualquer artigo que julgassem elegíveis. Também de maneira independente, revisaram e selecionaram ensaios a partir dos resultados das buscas, avaliando a adequação, a metodologia e a qualidade dos estudos. Os casos de discordância ou incerteza foram resolvidos por consenso ou por meio de consulta a um dos outros autores. 

### Extração dos dados

Os dados foram extraídos por meio de um protocolo adaptado da declaração *Preferred Reporting Items for Systematic Reviews and Meta-Analyses* (PRISMA, Itens Preferenciais para Relato em Revisões Sistemáticas e Meta-Análises),^(^ 13 ^)^ incluindo os dados de identificação do estudo; a duração do estudo; o desenho do estudo; critérios de inclusão e exclusão; critérios para o diagnóstico de asma; a idade e o gênero dos participantes; o número de participantes; o método de randomização; a gravidade da asma no(s) grupo(s) de estudo; os métodos de processamento e medição do escarro; e os métodos de avaliação de alterações pós-tratamento em eosinófilos, IL-5 e PCE no escarro, bem como no VEF_1_ pós-broncodilatador. 

### Análise estatística

Os dados foram analisados por meio do programa MIX para meta-análise, versão 1.7 (Centro de Pesquisa Clínica Kitasato, Sagamihara, Japão).^(^ 14 ^)^ Os estudos incluídos foram agrupados para produzir as médias ou medianas de eosinófilos no escarro, PCE no escarro, IL-5 no escarro e VEF_1_, com os respectivos IC95% ou ep. Para as variáveis contínuas, foram calculadas as médias e IC95%. Se os autores haviam relatado os dp, estes eram usados para calcular os ep, por meio da seguinte fórmula: 

*dp* = ep* √^(N)^

Quando os dp não estavam disponíveis para essas variáveis, os IC95% eram transformados em dp, por meio da seguinte fórmula: 

*ep* = (*limite superior do IC95% *−* limite inferior do IC95%*)/(1,96*2), *dp* = ep* √^(N)^.

A inconsistência entre as estimativas combinadas foi quantificada com a estatística I^2^ de Higgins, que mede o grau de heterogeneidade verdadeira e é determinada por meio da seguinte equação: 

*I2* = [(*Q* − *gl*)/*Q*] × 100

na qual *Q* é o Q de Cochran (baseado no qui-quadrado) e *gl* significa graus de liberdade. Isso ilustra a porcentagem de variabilidade em estimativas de efeitos resultantes da heterogeneidade, e não de um erro amostral. ^(^ 15 ^)^ Em caso de heterogeneidade, usava-se um modelo de efeitos aleatórios. Foram realizadas análises de sensibilidade em que se compararam os modelos de efeitos aleatórios e fixos. O potencial de viés de publicação foi avaliado por meio do teste de Egger, da estatística I^2^ de Higgins e de gráficos de funil (*funnel plots*). Um modelo de efeitos aleatórios foi usado para a análise de eosinófilos, IL-5 e PCE no escarro, em virtude da grande heterogeneidade desses marcadores nos estudos, sendo Q = 168,1; p < 0,001; I^2^ = 96,4%, Q = 8,7; p = 0,013; I^2^ = 77,0% e Q = 700,9; p < 0,001; I^2^ = 99,6%, respectivamente. 

## Resultados

Por meio das buscas nos bancos de dados, foram identificados 223 artigos. A revisão dos títulos e resumos resultou na exclusão de 191 estudos (Figura 1). Dos 32 artigos remanescentes, alguns foram ainda excluídos pelos seguintes motivos: ausência de informações a respeito de desfechos primários (n = 4)^(^ 6 ^,^ 16 ^)^; artigo de revisão ou meta-análise (n = 2)^(^ 17 ^,^ 18 ^)^; relato de caso ou série de casos (n = 3)^(^ 7 ^,^ 19 ^,^ 20 ^)^ e ausência de dados adequados para a meta-análise (n = 9; avaliação de um medicamento distinto, ausência de grupo controle ou ausência de dados pré e pós-tratamento referentes ao uso de prednisona ou prednisolona).^(^ 12 ^,^ 21 ^-^ 28 ^)^ Os 14 artigos remanescentes foram revisados, e apenas 8 preencheram os critérios de inclusão.^(^ 8 ^,^ 10 ^,^ 29 ^-^ 34 ^)^ As características dos estudos incluídos estão descritas na Tabela 1. 

**Figura 1:** **Fluxograma da seleção de estudos. PCE: proteína catiônica eosinofílica.** PCE: proteína catiônica eosinofílica.

**Tabela 1:** Características dos estudos incluídos.

Referência	Características do estudo	Tratamento	Pré-tratamento vs. pós-tratamento
Baigelman et al.^(30)^	Desenho: observacional	Medicamento: prednisona	Eosinófilos no escarro, média ± dp: 26,0 ± 19,0% vs. 8,0 ± 5,0%
	Amostra: 11 pacientes com asma (3 homens e 8 mulheres; 29-60 anos de idade) acompanhados durante pelo menos 4 anos	Dose: 80 mg/dia	PCE no escarro: [não medida]
			VEF_1_ pós-broncodilatador, média ± dp: 1,03 ± 0,41 L vs. 1,32 ± 0,51 L
	Perfil: exacerbação da asma	Duração: 3 dias	IL-5: [não medida]
Claman et al.^(31)^	Desenho: ensaio controlado randomizado		
	Amostra: 24 pacientes com asma — grupo prednisona oral (n = 12, 7 homens e 5 mulheres) e grupo placebo (n = 12, 6 homens e 6 mulheres) — sendo excluídos os pacientes que usaram corticosteroides inalatórios ou orais nas 6 semana anteriores, os que apresentaram infecção do trato respiratório superior nas 6 semana anteriores e os que apresentavam carga tabágica > 10 anos-maço	Medicamento: prednisona oral	Eosinófilos no escarro, média ± ep: 14,1 ± 5,0% vs. 1,8 ± 0,8%
		Dose: 0.5 mg/kg por dia	PCE no escarro, média ± ep: 325 ± 131 μg/mL vs. 144 ± 84 μg/mL
	Perfil: exacerbação da asma	Duração: 6 dias	VEF_1_ pós-broncodilatador, média ± dp: 88 ± 5,2% vs. 91 ± 4,87%
			IL-5: [não medida]
Keatings et al.^(34)^	Desenho: estudo cruzado simples-cego	Medicamento: prednisolona	Eosinófilos no escarro, média ± dp: 6,66 ± 0,98% vs. 0,99 ± 0,25%
	Amostra: 15 pacientes com DPOC (excluídos desta meta-análise) e 11 pacientes com asma atópica leve (1 excluído; 10 incluídos para análise; média de idade, 29,8 ± 3,4 anos)	Dose: 30 mg/dia	PCE no escarro, média ± dp: 687,0 ± 87,0 μg/L vs. 80,2 ± 14,2 μg/L
		Duração: 2 semanas	VEF_1_ pós-broncodilatador:
	Perfil: asma estável		-em %, média ± dp: 95,9 ± 5,7% vs. [não descritos]
			-em L, média ± ep: [não descritos] vs. 3,92 ± 0,32 L
			IL-5: [não medida]
Pizzichini et al.^(8)^	Desenho: observacional	Medicamento: prednisona	Eosinófilos no escarro, média ± dp: 17,6 ± 10,0% vs. 0,89 ± 0,90%
	Amostra: 10 pacientes com asma	Dose: 30 mg/dia durante 5 dias, reduzida para zero até o 10º dia	PCE no escarro, média ± dp: 5.338,4 ± 6.690,7 μg/L vs. 985,5 ± 1.311,0 μg/L
	Perfil: exacerbação da asma	Duração: 10 dias	VEF_1_ pós-broncodilatador, média ± dp: 1,5 ± 0,3 L vs. 2,5 ± 0,5 L
			IL-5, média ± dp: 201 ± 128 pg/mL vs. 0,0 ± 55,6 pg/mL
Pizzichini et al.^(10)^	Desenho: observacional	Medicamento: prednisona	Eosinófilos no escarro, média ± dp: 16,3 ± 32,3% vs. 0,0 ± 0,5%
	Amostra: 8 pacientes com asma dependente de prednisona; > 12% de variabilidade do VEF_1_, média basal, 18,5% (variação, 13-27%)	Dose: 30 mg/dia	PCE no escarro, média ± dp: 7.480 ± 5.240 μg/L vs. 700 ± 784 μg/L
	Perfil: asma grave	Duração: 7 dias	VEF_1_ pós-broncodilatador, média ± dp: 55,7 ± 6,84% vs. 80,0 ± 15,91%
			IL-5, média ± dp: 66,5 ± 150 pg/mL vs. 44,1 ± 86 pg/mL
Di Franco et al.^(32)^	Desenho: ensaio controlado randomizado	Medicamento: prednisona	Eosinófilos no escarro: 52,0% [sem dp ou ep] vs. 11,0% [sem dp ou ep]
	Amostra: 40 adultos não fumantes (9 homens e 31 mulheres; média de idade, 45 ± 13 anos; 3 excluídos), em dois braços — fluticasona (1.000 μg/dia; n = 18) e prednisona (n = 19)	Dose: 40 mg/dia, reduzida para 10 mg/dia por meio da redução de 5 mg da dose dia sim, dia não	PCE no escarro: 904 μg/L [sem dp ou ep] vs. [não descrita]
	Perfil: exacerbação da asma	Duração: 6 dias	VEF_1_ pós-broncodilatador, média ± dp: 51,5 ± 14,4% vs. 83,6 ± 21,1%
			IL-5: [não medida]
Scheicher et al.^(33)^	Desenho: observacional controlado	Medicamento: prednisona oral	-
	Amostra: 51 indivíduos — 21 indivíduos normais e 30 pacientes com asma (13 homens e 17 mulheres; média de idade, 41 anos), 9 dos quais jamais haviam tomado esteroides; estes receberam prednisona oral e foram avaliados antes e depois do tratamento	Dose: 40 mg/day	
	Perfil: asma estável	Duração: 14 dias	
Dente et al.^(29)^	Desenho: ensaio controlado randomizado	Medicamento: prednisona oral	-
	Amostra: 59 pacientes com asma refratária grave, aleatoriamente divididos em dois grupos, isto é, prednisona (n = 39) e placebo (n = 20)	Dose: 0,5 mg/kg por dia	
	Perfil: asma grave	Duração: 2 semanas	

PCE: proteína catiônica eosinofílica.

### Efeitos sobre eosinófilos, IL-5 e PCE no escarro

A análise conjunta (n = 198) revelou uma redução média de seis vezes no número de eosinófilos no escarro após o tratamento (↓8,2%; IC95%: 7,7-8,7; p < 0,001; Figura 2). Dentre os estudos que avaliaram IL-5 (em pg/mL) antes e depois do tratamento com prednisona ou prednisolona (n = 114), houve uma redução média de aproximadamente quatro vezes nos níveis de IL-5 (↓83,6; IC95%: 52,5-83,6; p < 0,001; Figura 3). Além disso, dentre os estudos que avaliaram a PCE (em μg/L) em indivíduos tratados com prednisona ou prednisolona (n = 80), o tratamento resultou em uma redução média de cinco vezes nos níveis de PCE (↓267,6; IC95%: 244,6-290,6; p < 0,001; Figura 4). 

**Figura 2:** Redução de eosinófilos no escarro após tratamento com prednisona ou prednisolona por via oral.

**Figura 3:** Redução dos níveis de IL-5 no escarro após tratamento com prednisona.

**Figura 4:** Redução dos níveis de proteína catiônica eosinofílica no escarro após tratamento com prednisona ou prednisolona por via oral.

### Efeitos sobre o VEF_1_

Foram também analisadas as mudanças no VEF_1_ pós-broncodilatador após o tratamento com prednisona ou prednisolona nos 194 pacientes com asma para os quais estavam disponíveis os dados relevantes.^(^ 8 ^,^ 10 ^,^ 29 ^-^ 32 ^)^ Nessa análise, foi também usado um modelo de efeitos aleatórios, em virtude da grande heterogeneidade (Q = 46,03; p < 0,0001; I^2^ = 89,1%). Após 6-14 dias de tratamento, houve um aumento médio significativo no VEF_1_ pós-broncodilatador (↑8,1%; IC95%: 5,3-10,8; z = 5,8; p < 0,001; Figura 5). Uma análise dos dados referentes aos valores absolutos de VEF_1_ pós-broncodilatador (em litros) revelou um aumento médio pós-tratamento, de 1,88 para 2,34 L (↑0,46 L; p < 0,001; dados não apresentados). 

**Figura 5:** Aumento do VEF1 pós-broncodilatador (% do previsto) após tratamento com prednisona.

### Gerenciamento de resultados

Em virtude da grande heterogeneidade, foi realizada uma metarregressão para examinar os efeitos do tratamento com prednisona ou prednisolona por idade, gênero e dose (Figura 6). A dose de prednisona pareceu ser responsável pela heterogeneidade dos níveis de eosinófilos no escarro (T^2^ = 8,753) e de PCE no escarro (T^2^ = 172,8). A regressão linear não mostrou nenhuma associação entre a dose de prednisona e eosinófilos no escarro (p = 0,55), PCE no escarro (p = 0,38), IL-5 no escarro (p = 1,00) ou VEF_1_ pós-broncodilatador (p = 0,27). 

**Figura 6:** Gráficos de funil (funnel plots). MD: mediana; PCE: proteína catiônica eosinofílica; e BD: broncodilatador.

## Discussão

Nossas análises mostram que o tratamento com prednisona ou prednisolona é altamente eficaz na redução de eosinófilos no escarro na bronquite eosinofílica. Essa redução é acompanhada de uma redução de outros marcadores inflamatórios no escarro ligados à bronquite eosinofílica, tais como PCE e IL-5. Além disso, constatou-se que o tratamento da bronquite eosinofílica com prednisona ou prednisolona resulta em um aumento significativo do VEF_1_ pós-broncodilatador. 

Dados sugerem que a eosinofilia no escarro e altos níveis de marcadores eosinofílicos estão relacionados com mau controle da asma, e não com a gravidade da asma.^(^ 7 ^,^ 8 ^,^ 21 ^,^ 26 ^)^ O componente da asma que responde aos corticosteroides é a bronquite eosinofílica, que pode agora ser reconhecida pelo confiável método da contagem de células em amostras de escarro induzido.^(^ 8 ^)^ O tratamento adequado com corticosteroides reduz a proporção de eosinófilos no escarro a valores normais, mesmo na asma dependente de prednisona.^(^ 8 ^,^ 19 ^)^ Nesta meta-análise, houve uma redução de seis vezes dos eosinófilos no escarro após o tratamento com prednisona ou prednisolona. 

Como a redução da eosinofilia no escarro está relacionada com uma resposta clínica e funcional positiva aos corticosteroides, nossos resultados apoiam a recomendação de que se deve aumentar a dose de corticosteroides quando a asma se torna descontrolada.^(^ 8 ^,^ 21 ^)^ Em uma análise de regressão múltipla, ten Brinke et al.^(^ 23 ^)^ constataram que o fator mais importante potencialmente relacionado com obstrução persistente do fluxo aéreo na asma grave foi uma proporção de eosinófilos no escarro > 2% (OR ajustada = 7,7). No entanto, existem indivíduos com asma não controlada que não apresentam eosinofilia no escarro. Ainda não se determinou se esse subgrupo é menos sensível aos corticosteroides. 

Os eosinófilos há muito são considerados mediadores inflamatórios essenciais na patogênese da asma, embora seu papel exato não esteja claro. Em estudos de populações heterogêneas de pacientes com asma, a regulação negativa da atividade de eosinófilos por meio da inibição específica de IL-5 (uma citocina pró-eosinofílica) produziu resultados decepcionantes.^(^ 35 ^)^

Em um estudo com pacientes com asma grave e inflamação eosinofílica das vias aéreas refratária,^(^ 22 ^)^ constatou-se que a administração de triancinolona por via intramuscular reduziu a proporção média de eosinófilos no escarro de 12,6% para 0,2%, semelhante às reduções obtidas por meio do uso de corticosteroides inalatórios (> 1.600 μg/dia) ou do uso crônico de prednisona oral.^(^ 29 ^,^ 32 ^)^ Os autores também observaram um aumento do VEF_1_ e uma redução do uso de medicação de resgate. ^(^ 22 ^)^ Esses dados estão de acordo com os de outros estudos com pacientes com asma e confirmam que a eosinofilia no escarro é um bom preditor da resposta aos corticosteroides.^(^ 6 ^-^ 8 ^,^ 36 ^)^ Não se sabe o motivo desse distinto efeito em curto prazo dos corticosteroides, em oposição ao bem conhecido efeito positivo em longo prazo na maioria dos pacientes com asma.^(^ 29 ^)^ Uma possível explicação é que a inflamação não eosinofílica talvez responda aos corticosteroides mais lentamente do que a inflamação eosinofílica.^(^ 37 ^)^

Os dados aqui apresentados ratificam a utilidade da avaliação de eosinófilos no escarro para prever quando pacientes com asma grave podem se beneficiar de um aumento da dose de corticosteroides.^(^ 6 ^-^ 8 ^,^ 29 ^,^ 36 ^)^ A classificação de fenótipos de asma grave em eosinofílicos e não eosinofílicos pode ter implicações clínicas na escolha da terapia farmacológica.^(^ 7 ^,^ 19 ^,^ 29 ^)^

A persistência de eosinofilia na asma grave poderia ser um reflexo da insensibilidade aos corticosteroides, e é possível que a asma refratária responda à terapia anti-IL-5 com mepolizumabe. ^(^ 4 ^,^ 5 ^,^ 38 ^,^ 39 ^)^ Como mencionado anteriormente, constatou-se no presente estudo que os níveis de IL-5 no escarro diminuíram após tratamento com prednisona ou prednisolona. Como a IL-5 é uma citocina pró-inflamatória que aumenta o recrutamento, a ativação e a sobrevivência de eosinófilos, é considerada de importância fundamental na fisiopatologia da asma.^(^ 40 ^)^ Ying et al.^(^ 41 ^)^ mostraram elevada expressão de IL-5 em células T, eosinófilos e mastócitos em amostras de biópsia brônquica de pacientes com asma. Outros estudos mostraram que os níveis de IL-5 no escarro tendem a ser maiores em pacientes com asma eosinofílica, ao passo que os de IL-8 tendem a ser maiores em pacientes com asma não eosinofílica.^(^ 29 ^,^ 42 ^)^ Há evidências de que a IL-5 é detectável no escarro induzido de pacientes com asma e que os níveis de IL-5 no escarro são mais elevados em pacientes com asma grave do que naqueles com asma de leve a moderada.^(^ 10 ^,^ 42 ^)^

Nossos resultados mostram que os corticosteroides sistêmicos são eficazes não só na redução dos níveis de eosinófilos no escarro, mas também na inibição da liberação de citocinas pró-inflamatórias que desempenham um papel relevante na perpetuação da inflamação das vias aéreas em pacientes com asma refratária. Nossos dados estão de acordo com os de outros estudos que mostram que os corticosteroides diminuem o número de células T ativadas que expressam RNA mensageiro de IL-4 e IL-5 no lavado broncoalveolar de pacientes com asma, independentemente da gravidade da asma.^(^ 43 ^)^ Nossos resultados também mostram que o tratamento com prednisona ou prednisolona pode resultar em uma redução de quatro vezes nos níveis de PCE no escarro. Quando consideramos os níveis de PCE em sobrenadantes de escarro, constatamos que esses níveis relacionaram-se com mau controle da asma, o que sublinha o fato de que os eosinófilos desempenham um papel nessa equação.^(^ 28 ^)^ No entanto, não obstante as expectativas de que os pacientes com asma grave apresentariam níveis mais elevados de PCE no escarro, Romagnoli et al.^(^ 21 ^)^ não observaram diferenças entre grupos de pacientes com asma, estratificados por gravidade da asma, no tocante aos níveis de PCE no escarro. 

Em um estudo de exacerbações agudas da asma, Baigelman et al.^(^ 30 ^)^ demonstraram que o VEF_1_ melhora dentro das primeiras 24 h de tratamento com prednisona ou prednisolona, e mais ainda após 48-72 h de tratamento. No entanto, em um estudo semelhante, Belda et al.^(^ 44 ^)^ não observaram nenhuma mudança no VEF_1_ nas primeiras 24 h. Aggarwal & Bhoi^(^ 12 ^)^ também estudaram exacerbações agudas da asma e sugeriram que metilprednisolona intravenosa seguida de metilprednisolona oral é um regime terapêutico mais eficaz e mais seguro do que hidrocortisona intravenosa seguida de prednisolona oral. No entanto, os autores empregaram apenas avaliação clínica e espirometria, sem analisar os mediadores inflamatórios no escarro ou outras secreções respiratórias. 

Em uma meta-análise realizada em 1992, Rowe et al.^(^ 17 ^)^ mostraram que o uso de corticosteroides no início do tratamento de exacerbações da asma reduz o número de internações hospitalares em adultos e crianças e que os corticosteroides são eficazes na prevenção de recidiva no tratamento ambulatorial de exacerbações da asma. Corticosteroides orais e intravenosos parecem ter efeitos equivalentes sobre a função pulmonar em exacerbações agudas,^(^ 17 ^)^ e estudos recentes demonstraram que corticosteroides inalatórios e orais são igualmente eficazes.^(^ 24 ^,^ 32 ^,^ 33 ^,^ 44 ^)^

Não obstante a heterogeneidade dos estudos aqui avaliados no tocante às doses e à duração do tratamento/acompanhamento,^(^ 8 ^,^ 10 ^,^ 29 ^-^ 31 ^)^ a maior parte da exposição a prednisona ou prednisolona foi > 30 mg/dia, o que é suficiente para suprimir os eosinófilos no escarro, reduzir os níveis de PCE, inibir a IL-5 e aumentar o VEF_1_. No entanto, em virtude da grande heterogeneidade, foi necessário realizar uma metarregressão de ajuste para a dose e duração do tratamento. 

A meta-análise é uma ferramenta poderosa para estudar dados cumulativos de estudos individuais com amostras pequenas e baixo poder estatístico. O agrupamento de efeitos de estudos individuais por meio de meta-análise pode aumentar o poder estatístico e pode ajudar a detectar diferenças modestas de risco entre grupos experimentais. 

É possível obter uma alteração detectável em índices inflamatórios durante 14 dias de tratamento com corticosteroides. Benefícios clínicos e efeitos anti-inflamatórios foram relatados em pacientes com asma tratados com esses esquemas curtos, que são comumente usados na prática clínica.^(^ 12 ^,^ 34 ^)^

Em suma, constatou-se que, em pacientes com bronquite eosinofílica de moderada a grave, o tratamento com prednisona ou prednisolona resultou em uma redução significativa dos níveis de eosinófilos no escarro, bem como dos níveis de IL-5 e PCE no escarro. Essa redução da resposta inflamatória foi acompanhada de um aumento significativo do VEF_1_ pós-broncodilatador.
